# Physical Activity Related to Body Muscle Mass Index and Stiffness Index in 7-to-10-Year-Old Girls

**DOI:** 10.3390/healthcare10020197

**Published:** 2022-01-20

**Authors:** Yingzhi Gu, Tadashi Ito, Yuji Ito, Koji Noritake, Nobuhiko Ochi, Naomichi Matsunaga, Daiki Takahashi, Hideshi Sugiura

**Affiliations:** 1 Department of Integrated Health Sciences, Graduate School of Medicine, Nagoya University, Nagoya 461-8673, Japan; gu.yingzhi@a.mbox.nagoya-u.ac.jp (Y.G.); sanjigen@mikawa-aoitori.jp (T.I.); matsunaga.naomichi@icloud.com (N.M.); takahashi.daiki@g.mbox.nagoya-u.ac.jp (D.T.); 2 Three-Dimensional Motion Analysis Room, Aichi Prefectural Mikawa Aoitori Medical and Rehabilitation Center for Developmental Disabilities, Okazaki 444-0002, Japan; 3 Department of Pediatrics, Aichi Prefectural Mikawa Aoitori Medical and Rehabilitation Center for Developmental Disabilities, Okazaki 444-0002, Japan; yuji.ito@med.nagoya-u.ac.jp (Y.I.); aoi2pochi@yahoo.co.jp (N.O.); 4 Department of Orthopedic Surgery, Aichi Prefectural Mikawa Aoitori Medical and Rehabilitation Center for Developmental Disabilities, Okazaki 444-0002, Japan; noritake@mikawa-aoitori.jp

**Keywords:** MVPA, pre-puberty girls, body muscle mass, bone mineral density, muscle strength

## Abstract

The relationship between moderate-to-vigorous physical activity (MVPA) performance time and body muscle mass and stiffness index in pre-puberty school-aged girls has not been fully elucidated. The effect of sexual maturity on bone mass is more pronounced in girls. This study aimed to clarify the relationship between MVPA performance time and the above-mentioned factors. This was a prospective, population-based cohort study of 111 girls aged 7–10 years. Data were collected via medical examination, clinical measurements, and questionnaires. Spearman’s ρ analysis was used to determine the association between skeletal muscle mass index (SMI) and stiffness index, muscle strength, and MVPA performance time. Participants who met the recommended MVPA level accounted for only 24.3% (*n* = 27) of all participating girls (*n* = 111). The following factors were significantly positively correlated with MVPA level at spearman’s ρ analysis: SMI (*r* = 0.303, *p* = 0.001), stiffness index (*r* = 0.229, *p* = 0.015), grip strength (*r* = 0.283, *p* = 0.003), back muscle strength (*r* = 0.197, *p* = 0.038), and standing long jump distance (*r* = 0.288, *p* = 0.002). Multiple regression analysis’s results revealed that SMI (*β* = 0.237; *p* = 0.024) was associated with MVPA performance time. These results can help school-aged girls to pay adequate attention to having healthy physical activity habits to prevent the decline of skeletal muscle mass, stiffness index, and body muscle strength.

## 1. Introduction

In recent years, regular physical activity has been regarded as an essential factor of healthy living habits. According to the World Health Organization, regular physical activity can prevent cardiovascular disease, reduce the risk of obesity, and establish a good mental state [1]. Moreover, compared to mild physical activity, such as walking, moderate to vigorous physical activity is better for both physical and mental health [2]. The guidelines established by Prochaska et al. stated that children are recommended to perform moderate to vigorous physical activity (MVPA) for at least 60 min every day [3]. Low MVPA performance has been associated with higher cardiometabolic risk factors [4]. At the same time, physical activity intervention can also reduce the risk of cardiovascular disease in overweight girls [5].

Previous cross-sectional studies among elementary school children showed that boys engage in more moderate to vigorous physical activity on weekends than girls, and seniors have significantly less physical activity [6]. Factors such as sex, dietary intake, obesity, sexual maturity, sedentary habits, and sleep duration are all thought to be associated with MVPA compliance [7]. Grip strength is a very crucial indicator because it predicts the total muscle strength [8]. It is understood that the back muscle should be studied as the largest trunk muscle in the human body, the burst of lower extremities muscle strength is also worth measuring as a predictor of muscle fitness [9]. Skeletal muscles are considered to be one of the most important components of the body and play an important role in movement and postural maintenance as well as insulin-mediated glucose disposal [10,11]. Muscle fitness also plays a relevant role in physical activity, especially vigorous-intensity activity [12]. Therefore, due to long-term differences in the amount of exercise, differences in muscle strength and body composition such as skeletal muscle mass and bone mineral density between the children performing the recommended MVPA level and the children performing substandard MVPA levels need to be reported. Vigorous-intensity physical activity interventions have been shown to have a positive effect on skeletal muscle mass and bone mineral density in children, but the effects of exercise on girls are less pronounced than in boys [13]. Girls with low MVPA levels may experience health problems, such as fractures and pubertal development problems. There was also shown that girls who took part in pre-pubertal gymnastics had better bone health than those who did not [14]. Therefore, it may be valuable to identify signs of declining bone density and muscle function in girls who do not undergo MVPA. A follow-up studies have shown that physical activity could increase spine bone mass and muscle strength in children [15]. Children with low MVPA levels may experience health problems later in their lives, on account of there is now epidemiological evidence that children are prone to sarcopenia and chronic diseases [16].

The purpose of this study was to clarify the relationship between MVPA performance time and body composition such as skeletal muscle mass, stiffness index, which is a reliable tool for measuring total body’s bone development measured by ultrasound device and body muscle strength, including grip, back, and leg muscle strength in girls between the ages of 7 and 10 years. Considering the disparity in the intensity and frequency of physical activity between boys and girls and the increased rate of bone mineral density development in girls during puberty [17], the influence of sex and age on bone mineral density and exercise time needs to be considered. Hence, pre-puberty elementary school girls in the lower and middle grades were included in our study. We hypothesized that school girls aging between 7 and 10 years and performing the recommended MVPA level have higher body muscle mass index, stiffness index, and skeletal muscle strength.

## 2. Materials and Methods

### 2.1. Participants

A population-based cohort study (“Okazaki child medical check-up for physical function”) was performed between January 2018 and March 2020. Elementary school children from Okazaki city aged 7–10 years were selected to participate in the medical check-up study. The medical checkup consisted of a medical examination by a pediatric orthopedic surgeon and a pediatric neurologist; clinical measurements including body composition and muscle strength by trained pediatric physiotherapists; and questionnaires regarding time of physical activity were assessed. The exclusion criteria were as follows: (1) inability to complete the medical check-up; (2) orthopedic, neurological, ophthalmologic, auditory, respiratory, or cardiovascular abnormalities that may affect the results of clinical measurements; (3) previously diagnosed developmental disabilities such as autism spectrum disorder or attention deficit hyperactivity disorder; (4) substandard score for the Raven’s Colored Progressive Matrices; and [18] the Picture Vocabulary Test-Revised (Nihon Bunka Kagakusha Co., Ltd., Tokyo, Japan), which indicates intellectual disabilities. If one of the exclusion criteria was met, the participant will be eliminated. Therefore, 152 children were excluded, and 285 boys and girls were enrolled. To focus on the relationship between MVPA performance times, body composition such as muscle mass and stiffness index, and body muscle strength in pre-puberty girls, we excluded all the boys and the girls who may have reached puberty. Eventually, 111 girls were enrolled in this study as participants. Written informed assent and informed consent were obtained from all participants and their parents, respectively, prior to participants’ inclusion in the study, and this research has been approved by the institutional review board of the authors’ affiliated institutions. The manuscript was prepared in accordance with the Strengthening the Reporting of Observational Studies in Epidemiology (STROBE) guidelines.

### 2.2. Data Collection

#### 2.2.1. Grouping Based on Physical Activity

The World Health Organization has recommended at least 60 min/day of moderate-to-vigorous physical activity (MVPA) performance times for school-age children [19]. The Japanese version of the World Health Organization health behavior questionnaire for school-aged children has also been proven reliable [20]. The MVPA performance time of the participants in this study was collected using this questionnaire. Children who self-reported MVPA performance time of ≥ 60 min/day during a week were considered to meet the recommended MVPA level. < 60 min of MVPA time per day during a week were considered to perform substandard MVPA level.

#### 2.2.2. Body Composition Measurements

(1) Skeletal muscle mass index (SMI)

A multi-frequency bioelectrical impedance analyzer (MC-780A, Tanita, Tokyo, Japan) was used to measure the skeletal muscle mass index (SMI) [21]. First, the staff input basic information including height, age, and sex. The participants were then asked to stand on the platform with the anterior and posterior feet electrodes by themselves while grabbing the hand electrodes, ensuring contact between the palm and thumb of both hands with the electrodes. Then, the arms were expanded on both sides. After a few seconds, the recorded SMI information was available.

(2) Stiffness index

An ultrasound heel bone measuring device A-1000 EXP II (GE Healthcare Japan, Tokyo, Japan) was used to measure bone mineral density. The parameters available from these measurements include broadband ultrasonic attenuation (BUA), which measures the frequency dependence of ultrasound attenuation and speed of sound (SOS), which reflects the transmission velocity of ultrasound passing through soft tissue [22]. Previous studies have shown that stiffness index is a reliable tool for measuring total body’s bone development during childhood and adolescence [23]. The stiffness index was calculated from these two parameters using the following equation: Stiffness index = (0.67 × BUA) + (0.28 × SOS) − 420 [24]. During the measurement, the participant’s right foot was placed in the test tank of the machine, and alcohol was sprayed on the ankle and on the contact surface of the machine for determination. The stiffness index was calculated and automatically recorded by the device.

#### 2.2.3. Body Muscle Strength Measurements

(1) Grip strength

We measured the children’s grip strength in kilograms using a Smedley-type handheld dynamometer (GRIP-D; Takei Ltd., Niigata, Japan), and took the average measurement of both hands. In the sitting position, the elbows were extended at 0°, the shoulders were in contact with the torso, and the wrists were kept in a neutral position to measure maximum grip strength. Verbal encouragement such as, “Keep it up” was provided during the assessment to maximize grip strength for 5 s [21].

(2) Back muscle strength

A digital back muscle dynamometer (Back-D; Takei Ltd., Niigata, Japan) was used to measure the participant’s back muscle strength [25,26]. The participants were asked to stand with their feet shoulder-width apart on a platform, and then adjust the chain length to accommodate their height differences. Back muscle strength was determined based on the maximum isometric extension strength of the trunk muscles in the standing position with 30° of trunk flexion [26]. This was to ensure that the force came from the back muscles, but not from the lower back muscle or shoulder muscles and took care to protect the participant from falling backward. We measured the data twice; the average of the two values was recorded.

(3) Standing long jump distance

We placed the tape measure parallel to the direction of the jump on the ground, perpendicular to the starting line to measure the distance of the standing long jump [9]. Participants placed their feet behind the starting line, with their feet shoulder-width apart. Swinging of arms and bending of knees were regularly instructed for better take-off. The participants attempted to jump as far as they could, landing with both feet touching the floor at the same time and without falling backward. The distance was measured from the takeoff line to the heel which landed closer to the takeoff line. We performed the tests twice; the maximum value of the two measurements was recorded.

### 2.3. Sample Size

The sample size for the multiple regression analysis was determined using a power analysis. Power analysis was conducted with G*Power (Heinrich Heine University of Düsseldorf, Düsseldorf, Germany) using an alpha of 0.05, a power of 0.8, a medium effect size (f^2^ = 0.15), and a number of predictors is 7 [27,28]. Based on these assumptions, the required sample size was determined to be 103.

### 2.4. Statistical Analysis

The normal distribution of each variable was confirmed using the Shapiro–Wilk test. Variable data for children performing the recommended MVPA level and children performing substandard MVPA level were compared using the independent t-test (after assessing variance using Levene’s test) or Mann–Whitney U test. Statistical significance was set at a *p* value < 0.05. Spearman’s rank correlation coefficient analysis was used to examine the association between SMI, stiffness index, grip strength, back muscle strength, standing long jump distance, and MVPA performance time. Log transformation was applied to validate the relation between MVPA and stiffness index, grip strength, back muscle strength, and standing long jump distance because both variables exhibited non-normal distributions. Next, multiple regression analysis was used to assess the relationship between physical activity and functional outcomes, and the results were after controlling for confounding factors (age and height). All data management and statistical computations were performed using the IBM SPSS Statistics software (version 24.0; SPSS Inc., Chicago, IL, USA). 

## 3. Results

From the results of this study, girls who met the recommended MVPA level accounted for only 24.3% (*n* = 27) of all participating girls (*n* = 111). The characteristics of the participants and comparisons between children performing the recommended MVPA level and performing the substandard MVPA level are summarized in Table 1. 

Variables with significant differences were SMI (*p* = 0.049), stiffness index (*p* = 0.025), grip strength (*p* = 0.041), and standing long jump distance (*p* = 0.039). Spearman’s rank correlation coefficient analysis revealed a significant positive correlation between MVPA performance time and SMI (*r* = 0.303, *p* = 0.001), stiffness index (*r* = 0.229, *p* = 0.015), grip strength (*r* = 0.283, *p* = 0.003), back muscle strength (*r* = 0.197, *p* = 0.038), and standing long jump distance (*r* = 0.288, *p* = 0.002) (Table 2).

Multiple regression analysis was used to analyze the correlation between children’s MVPA performance time and age, height, skeletal muscle mass index, stiffness index, grip strength, back muscle strength, and standing long jump distance. In all of these independent variables, only skeletal muscle mass index (*p* = 0.024) was turned out to have a significant relation with MVPA performance time (Table 3).

## 4. Discussion

This is the first study to identify the association between MVPA performance time and skeletal muscle mass index in prepubertal Japanese girls. In this population-based study, we demonstrated that MVPA performance time was positively associated with higher SMI and stiffness index, stronger grip strength and back muscle strength, and standing long jump distance in 7- to-10-year-old elementary school girls by spearman’s rank correlation coefficient analysis, while MVPA performance time was significantly related to skeletal muscle mass index at multiple regression analysis, which supported our hypothesis. Among all participants, only 24.3% of girls met the recommended MVPA level set by the World Health Organization in this survey. Moreover, the level of MVPA in female adolescents showed a declining trend with age [29]. This suggests that levels of physical activity are inadequate for girls before puberty and even throughout the age group. It reminds parents, schoolteachers, and children themselves to pay more attention to participate in adequate physical exercise in order to prevent the decline of skeletal muscle mass, stiffness index, and body muscle strength.

In the results from multiple regression analysis, the independent variable which turned out to be associated with MVPA performance time was skeletal muscle mass index only among girls aged 7–10 years old. This may imply that the reduction in MVPA performance time increases the risk of reduction in skeletal muscle mass index. It was also found in a previous study of Japanese children aged 6 to 12 that the skeletal muscle mass index was independently associated with moderate-to-vigorous physical activity [21]. Considering that skeletal muscle mass index is a crucial health measurement in adolescence and later life, 7- to 10-year-old elementary school girls should increase the duration of MVPA performance time appropriately in order to maintain skeletal muscle mass index. A future longitudinal study is needed to confirm these hypotheses. Skeletal muscle mass is not only critical in childhood and adolescence, but is also associated with sarcopenia in adulthood [10]. As a huge metabolic organ, skeletal muscle also plays a significant part in the decomposition of glucose, so skeletal muscle index can also reflect the metabolic health of the body [11,30]. What is more, since the duration of MVPA is significantly declined after girls reach puberty, physical activity intervention such as interview and feedback for girls before puberty is also essential [5]. Intervening in MVPA level from pre-puberty may prepare girls for healthier skeletal muscle mass and strong body later in life.

Our results indicate that skeletal muscle mass and stiffness index was positively correlated with MVPA performance time in pre-puberty schoolgirls. Previous studies have shown that levels of physical activity are positively correlated with skeletal muscle mass and stiffness index in a cohort of teenagers [21,31,32]. Another study reported that prospective exercise interventions increased skeletal muscle mass and bone mineral acquisition in premenarcheal girls [33]. In the current study, performing the recommended MVPA was also considered an important component of the body muscle mass and stiffness index status of 7- to 10-year-old girls. From a long-term perspective, it can be assumed that low body muscle mass and stiffness index values in puberty and adulthood are primarily caused by low physical activity levels in girls aged 7–10 years. Therefore, maintaining an optimal level of physical activity level during pre-puberty could improve body muscle mass and stiffness index later in life.

Muscle strength, including grip strength and the standing long jump distance, were highly proportional to MVPA performance time in pre-puberty elementary school girls. A prior study demonstrated that muscle strength was an independent predictor of stiffness index and body muscle mass in young women [34]. This suggests that girls with a good arm and lower leg strength have a habit of engaging in more MVPA and could maintain appropriate body muscle mass and stiffness index even during puberty and adulthood. Standing long jump value was considered an indicator of muscle health in adolescents [9], and grip strength has also been identified as a predictor of overall muscle strength [8]. Although back muscle strength did not show an obvious difference, it remained in direct proportion to MVPA performance time.

The present study has several limitations. First, we recruited participants from Okazaki city only. However, it is beneficial to investigate in a wider area, such as all over Japan or other countries, to generalize the findings. Second, moderate to vigorous physical activity is defined by measured value of METs, but because it is difficult to monitor in daily time; hence, data were collected using the Japanese version of the World Health Organization health behavior questionnaire for school-aged children [20]. Therefore, it is possible that the participants underestimated or overestimated their physical activity levels. Last, but not the least, we could not account for the role of dietary intake, which may be a confounding factor of body composition and muscle strength.

## 5. Conclusions

We found that school-aged girls who performed the recommended MVPA level were associated with having a higher SMI, stiffness index, grip strength, back muscle strength, and standing long jump distance, while skeletal muscle mass index was associated with MVPA performance time. These results can help not only school-aged girls themselves but also their parents and school managers to pay attention to having healthy physical activity habits to maintain good physical and mental health in later life.

## Figures and Tables

**Table 1:** Characteristics of participants.

Variables	Children Performing Recommended MVPA Level	Children Performing Substandard MVPA Level	*p*-Value
Number	27	84	
Age (year) ^a^	9 (7–10)	8 (7–10)	0.071
Height (cm) ^b^	129.7 ± 8.2	126.6 ± 7.4	0.071
Weight (kg) ^a^	25.4 (17.8–45.1)	25.0 (17.9–42.2)	0.232
Body mass index (kg/m^2^) ^a^	15.5 (13.0–23.6)	15.3 (13.1–22.8)	0.503
Skeletal muscle index (cm^2^/m^2^) ^b^	5.67 ± 0.44	5.47 ± 0.44	0.049 *
Stiffness index ^a^	83 (62–104)	78 (31–108)	0.025 *
Grip strength (kg) ^a^	11.3 (6.9–15.9)	10.3 (6.7–16.5)	0.041 *
Back muscle strength (kg) ^a^	27.8 (20.0–50.0)	27.3 (20.0–52.8)	0.392
Standing Long Jump distance (cm) ^a^	139 (114–160)	131 (83–185)	0.039 *
Physical activity time per week (h) ^a^	8 (7–18)	2.63 (0–6.83)	0.0001 *

^a^ Analysis result of using Mann–Whitney U-test. ^b^ Analysis result of using the independent *t*-test. * Significant at *p* < 0.05. MVPA, moderate-to-vigorous physical activity.

**Table 2:** Factors associated with time of performing moderate to vigorous physical activity among the all participants (*n* = 111) ^a^.

Variables	MVPA Performance Time	*p* Value
Skeletal muscle mass index	0.303	0.001 **
Stiffness index	0.229	0.015 *
Grip strength	0.283	0.003 **
Back muscle strength	0.197	0.038 *
Standing long jump distance	0.288	0.002 **

**^a^** Analysis result from spearman’s rank correlation coefficient analysis. * Significant at *p* < 0.05. ** Significant at *p* < 0.01. MVPA, moderate-to-vigorous physical activity.

**Table 3:** Multiple regression analysis with MVPA performance time as the dependent variable among the all participants (*n* = 111).

Independent Variables	Standardized β	*p* Value
Age	0.070	0.618
Height	−0.170	0.224
Skeletal muscle mass index	0.237	0.024 *
Stiffness index	0.112	0.299
Grip strength	0.115	0.354
Back muscle strength	−0.023	0.839
Standing long jump distance	0.200	0.098

The dependent variable was the MVPA performance time. The independent variables are skeletal muscle mass index, stiffness index, grip strength, back muscle strength, and standing long jump distance. The results were adjusted for age and height. * Significant at *p* < 0.05 MVPA, moderate-to-vigorous physical activity.

## Data Availability

All relevant data are presented within the manuscript. All data are available from the corresponding author on reasonable request.
